# In silico drug repurposing carvedilol and its metabolites against SARS-CoV-2 infection using molecular docking and molecular dynamic simulation approaches

**DOI:** 10.1038/s41598-023-48398-6

**Published:** 2023-12-04

**Authors:** Chunye Zhang, Jiazheng Liu, Yuxiang Sui, Shuai Liu, Ming Yang

**Affiliations:** 1 Christopher S. Bond Life Sciences Center, University of Missouri, Columbia, MO 65212 USA; 2 State Key Laboratory of Quality Research in Chinese Medicine, Macau Institute for Applied Research in Medicine and Health, Macau University of Science and Technology, Taipa, Macau, 999078 China; 3 School of Life Science, Shanxi Normal University, Linfen, 041004 Shanxi China; 4 The First Affiliated Hospital, Zhejiang University, Hangzhou, 310006 Zhejiang China; 5 Department of Surgery, University of Missouri, Columbia, MO 65212 USA; 6 NextGen Precision Health Institution, University of Missouri, Columbia, MO 65212 USA

**Keywords:** Bioinformatics, Antivirals, SARS-CoV-2

## Abstract

The pandemic of coronavirus disease 2019 (COVID-19) caused by the infection of severe acute respiratory syndrome coronavirus 2 (SARS-CoV-2) has caused a significant impact on the economy and public health worldwide. Therapeutic options such as drugs and vaccines for this newly emerged disease are eagerly desired due to the high mortality. Using the U.S. Food and Drug Administration (FDA) approved drugs to treat a new disease or entirely different diseases, in terms of drug repurposing, minimizes the time and cost of drug development compared to the de novo design of a new drug. Drug repurposing also has some other advantages such as reducing safety evaluation to accelerate drug application on time. Carvedilol, a non-selective beta-adrenergic blocker originally designed to treat high blood pressure and manage heart disease, has been shown to impact SARS-CoV-2 infection in clinical observation and basic studies. Here, we applied computer-aided approaches to investigate the possibility of repurposing carvedilol to combat SARS-CoV-2 infection. The molecular mechanisms and potential molecular targets of carvedilol were identified by evaluating the interactions of carvedilol with viral proteins. Additionally, the binding affinities of in vivo metabolites of carvedilol with selected targets were evaluated. The docking scores for carvedilol and its metabolites with RdRp were − 10.0 kcal/mol, − 9.8 kcal/mol (1-hydroxyl carvedilol), − 9.7 kcal/mol (3-hydroxyl carvedilol), − 9.8 kcal/mol (4-hydroxyl carvedilol), − 9.7 kcal/mol (5-hydroxyl carvedilol), − 10.0 kcal/mol (8-hydroxyl carvedilol), and − 10.1 kcal/mol (O-desmethyl carvedilol), respectively. Using the molecular dynamics simulation (100 ns) method, we further confirmed the stability of formed complexes of RNA-dependent RNA polymerase (RdRp) and carvedilol or its metabolites. Finally, the drug-target interaction mechanisms that contribute to the complex were investigated. Overall, this study provides the molecular targets and mechanisms of carvedilol and its metabolites as repurposed drugs to fight against SARS-CoV-2 infection.

## Introduction

The spread of severe acute respiratory syndrome coronavirus 2 (SARS-CoV-2) infection causes a global coronavirus disease 2019 (COVID-19) pandemic. According to the report of World Health Organization (WHO) (https://covid19.who.int/), there are 771,820,937 confirmed COVID-19 cases and 6,978,175 deaths among these cases worldwide by November 8, 2023. In May 2023, WHO announced the end of the COVID-19 emergency and started the transition to long-term management of COVID-19. There is an immeasurable economic cost related to COVID-19 during and post-pandemic periods. It is caused by an enveloped, single-stranded positive-sense RNA virus, belonging to the genus *Betacoronavirus*, one of the four coronaviral genera (*Alphacoronavirus* (α-), *Betacoronavirus* (β-), *Gammacoronavirus* (γ-), and *Deltacoronavirus* (δ-))^1–4^. Within the *Betacoronavirus*, SARS-CoV-2 shares 79% of sequence identity with SARS-CoV-1 and 50% of sequence similarity with MERS-CoV^5^. The genome of SARS-CoV-2 is 29.9 kb in length, which encodes structural proteins comprising spike protein (S), envelope protein (E), membrane protein (M), nucleocapsid protein (N), 16 non-structural proteins (NSP1-NSP16) in the open reading frame (ORF)1a and ORF1b regions, and 9 accessory proteins located in the region that contains the structural proteins^6–8^. S protein plays an essential role in the entry of SARS-CoV-2 into host cells, including subunits S1 and S2. Subunit S1 recognizes and binds with the human angiotensin-converting enzyme 2 (hACE2) receptor located on the surface of host epithelium cells in the lungs as well as blood vessels, then subunit S2 functions on the membrane fusion between the viral membrane and host cell membrane to finish the virus entry process^9^. Of note, there are two pathways of membrane fusion. One pathway is through the direct membrane fusion between the viral membrane and host cell membrane on the cell surface to release the viral genomic RNAs into the host cells, while the other pathway is processed by endocytosis to finish the membrane fusion inside the host cells and the release of viral RNAs^10,11^. After virus entry and membrane fusion, viral replication and proliferation proceed, then the newly made viruses are released from the infected host cells to infect other host cells.

Some important viral proteins can serve as therapeutic targets. Viral proteins including the structure and non-structure proteins play a pivotal role in the viral life cycle and pathogenesis^7,12^. The receptor binding domain (RBD) of the spike protein subunit S1 binds with human ACE2 to form the RBD-ACE2 complex facilitating the membrane fusion and virus entry into host cells. Based on this mechanism, a therapeutic agent that can inhibit or block the binding process by targeting the RBD-ACE2 complex is one of the therapeutic options to fight against SARS-CoV-2 infection^13,14^. The 3-chymotrypsin-like protease (3CLpro), also known as main protease (Mpro) or NSP5, is another favorable therapeutic target, which is associated with the release and maturation of NSP4-NSP16 and has the role in the cleavage of both virus and host proteins. 3CLpro also contributes to the interruption of host innate immunity^15,16^. Additionally, NSP12, well-known as RNA-dependent RNA polymerase (RdRp) that shares more than 95% sequence identified with SARS-CoV-1, can serve as a promising therapeutic target due to its close association with viral genome transcription and virus replication. This polymerase is the inhibitor target of the FDA-approved drug remdesivir (VEKLURY) for emergency application in SARS-CoV-2 treatment^17–20^. Papain-like protease (PLpro, a domain of Nsp3) is responsible for virus spread via the generation of replicase complex, a target for inhibiting viral transmission^21,22^. Furthermore, NSP13 helicase contributes the energy to facilitate the unwinding of RNA, which is critical to virus RNA replication and virus propagation^23,24^. NSP2 also plays an important role in facilitating virus replication by suppressing the production of immunomodulator IFN-β to impair host innate immunity to virus infection^25^. The above-mentioned proteins serve as ideal therapeutic drug targets for the treatment of COVID-19^26,27^.

A recent study discovered that the usage of carvedilol was associated with a lower rate of COVID-19-positive diagnosis. As a U.S. FDA-approved drug, carvedilol belongs to the third generation of beta-blockers, which has been used to treat hypertension due to its vasodilating properties^28^. In contrast to the traditional beta-blockers that can increase the expression of ACE2, carvedilol has the capability to reduce the expression of ACE. ACE serves as the binding receptor of SARS-CoV-2 on the membrane of host cells, facilitating virus entry into host cells. Therefore, the reduction of ACE expression level could limit virus binding and entry into host cells. Thus, carvedilol may inhibit SARS-CoV-2 infection by preventing the binding of viruses with host cells through inhibition of ACE expression^29^. In vitro assay also tested that carvedilol displayed strong anti-SARS-CoV-2 function in human lung epithelial cells. Metadata network analysis of virus protein-host protein interaction (PPI) revealed that carvedilol can serve as a potential drug for repurposing to treat COVID-19 due to its involvement in multiple important pathways, such as mRNA splicing and glucose metabolism^30^. In addition, carvedilol has the effect of reducing platelet aggregation^31^, which has been shown to be increased in COVID-19 patients in the intensive care unit (ICU)^32^. Thus, using carvedilol may contribute to the modulation of platelet levels in COVID-19 patients. Furthermore, it also has been suggested that carvedilol can regulate the expression of pro-inflammatory cytokines (e.g., IL-6) to reduce inflammation in COVID-19 patients^33–36^.

Although there are some beneficial effects of carvedilol against COVID-19, its anti-SARS-CoV-2 infection mechanism currently remains unclear. In this study, we first investigated the molecular mechanisms of carvedilol against SARS-CoV-2 infection using an in silico computational molecular docking approach. In addition, we further investigated the binding affinity of in vivo metabolites of carvedilol with SARS-CoV-2 virus, including 1-hydroxyl carvedilol, 3-hydroxyl carvedilol, 4-hydroxyl carvedilol, 5-hydroxyl carvedilol, 8-hydroxyl carvedilol, and O-desmethyl carvedilol. Finally, molecular dynamic simulation (MD) was employed to perform the in silico molecular simulation to study the stability of each drug-target complex and analyze the contact and interaction between small molecules and target proteins.

## Results

### In silico molecular docking of carvedilol with target proteins of SARS-CoV-2

**Figure 1:**
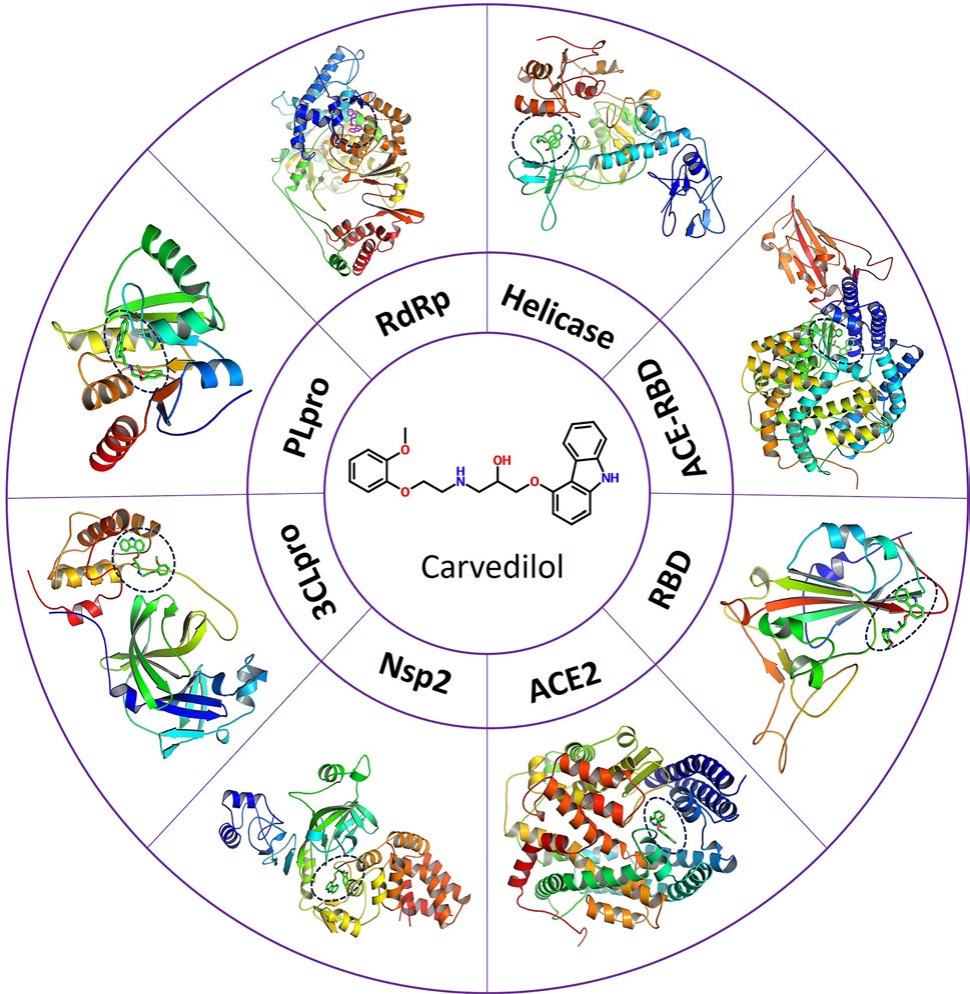
Schematic illustration of different SARS-CoV-2 proteins used to dock with carvedilol. Prepared protein structures used for docking are originally generated from the crystal structure obtained from the PDB database with details in the Methods. RdRp: RNA-directed RNA polymerase, Helicase: Non-structural protein 2 (NSP13), ACE2: angiotensin-converting enzyme 2, RBD: SARS-CoV-2 spike protein S1 receptor-binding domain, NSP2: Non-structural protein 2, 3CLpro/Mpro: main protease, PLpro: Papain-like protease (NSP3).

To investigate the potential mechanisms of carvedilol against SARS-CoV-2 infection, we first applied computational-based approaches to test the binding affinities of carvedilol with potential target proteins of SARS-CoV-2, including RdRp, 3CL/Mpro, NSP13 helicase, NSP2, PLpro, RBD, ACE2, and the complex of RBD-ACE2 (Fig. 1). To find the best protein–ligand binding complex with the highest binding affinity, we performed blind molecular docking using RdRp crystal structure (PDB ID:6XQB, resolution of 3.40 Å, Chain A) to dock with carvedilol and its metabolites. The molecular docking method allowed to screen the best binding sites of candidate compounds with target proteins. In silico molecular docking results showed a higher docking score (− 10.0 kcal/mol) between carvedilol and viral protein RdRp compared to the scores of other tested proteins, including 3CL/Mpro (− 7.2 kcal/mol), NSP13 Helicase (− 7.4 kcal/mol), NSP2 (− 7.5 kcal/mol), PLpro (− 7.9 kcal/mol), RBD (− 6.8 kcal/mol), ACE2(− 8.0 kcal/mol), and the complex of RBD-ACE2 (− 8.1 kcal/mol). This suggests that RdRp could be an ideal potential drug target for carvedilol against SARS-CoV-2 infection.

RdRp is also known as the drug target of remdesivir. Then, we used the same docking system to perform the molecular docking between remdesivir and RdRp. The molecular docking scores between remdesivir and carvedilol with RdRp were − 9.1 kcal/mol and − 10.1 kcal/mol, respectively, suggesting that carvedilol had a higher binding affinity with RdRp compared to remdesivir (Table 1).

**Table 1:** Molecular docking results for the binding affinity scores of viral target proteins and carvedilol and its metabolites.

Proteins targets/Molecules	RdRp (kcal/mol)	3CL/Mpro (kcal/mol)	Helicase/NSP13 (kcal/mol)	NSP2 (kcal/mol)	PLpro (kcal/mol)	RBD (kcal/mol)	ACE2 (kcal/mol)	RBD-ACE2 complex (kcal/mol)
Carvedilol	− 10.0	− 7.2	− 7.4	− 7.5	− 7.9	− 6.8	− 8.0	− 8.1
1-hydroxyl carvedilol	− 9.8	− 7.5	− 7.4	− 8.1	− 7.6	− 6.9	− 8.5	− 8.1
3-hydroxyl carvedilol	− 9.9	− 6.7	− 7.9	− 7.4	− 7.2	− 6.8	− 8.0	− 8.2
4-hydroxyl carvedilol	− 9.8	− 7.1	− 7.2	− 8.1	− 8.2	− 6.9	− 8.0	− 8.4
5-hydroxyl carvedilol	− 9.7	− 7.1	− 7.8	− 7.9	− 7.7	− 6.9	− 8.2	− 8.2
8-hydroxyl carvedilol	− 10.0	− 7.3	− 7.5	− 8.3	− 7.7	− 7.1	− 7.8	− 8.1
O-Desmethyl carvedilol	− 10.1	− 7.3	− 7.3	− 7.6	− 8.0	− 6.9	− 8.5	− 8.3
Remdesivir	− 9.1	− 7.9	− 8.7	− 8	− 8.7	− 7.2	− 8.3	− 8.7

Molecular docking results of binding affinity between each target viral protein with carvedilol and its metabolites (1-hydroxyl carvedilol, 3-hydroxyl carvedilol, 4-hydroxyl carvedilol, 5-hydroxyl carvedilol, 8-hydroxyl carvedilol, and O-desmethyl carvedilol), as well as the control remdesivir, showed that different molecules had a higher binding affinity with viral RdRp compared to the other tested viral proteins (3CL/Mpro, helicase, NSP2, PLpro, RBD, ACE2, RBD-ACE2 complex).

### In silico molecular docking of carvedilol metabolites with target viral proteins of SARS-CoV-2

**Figure 2:**
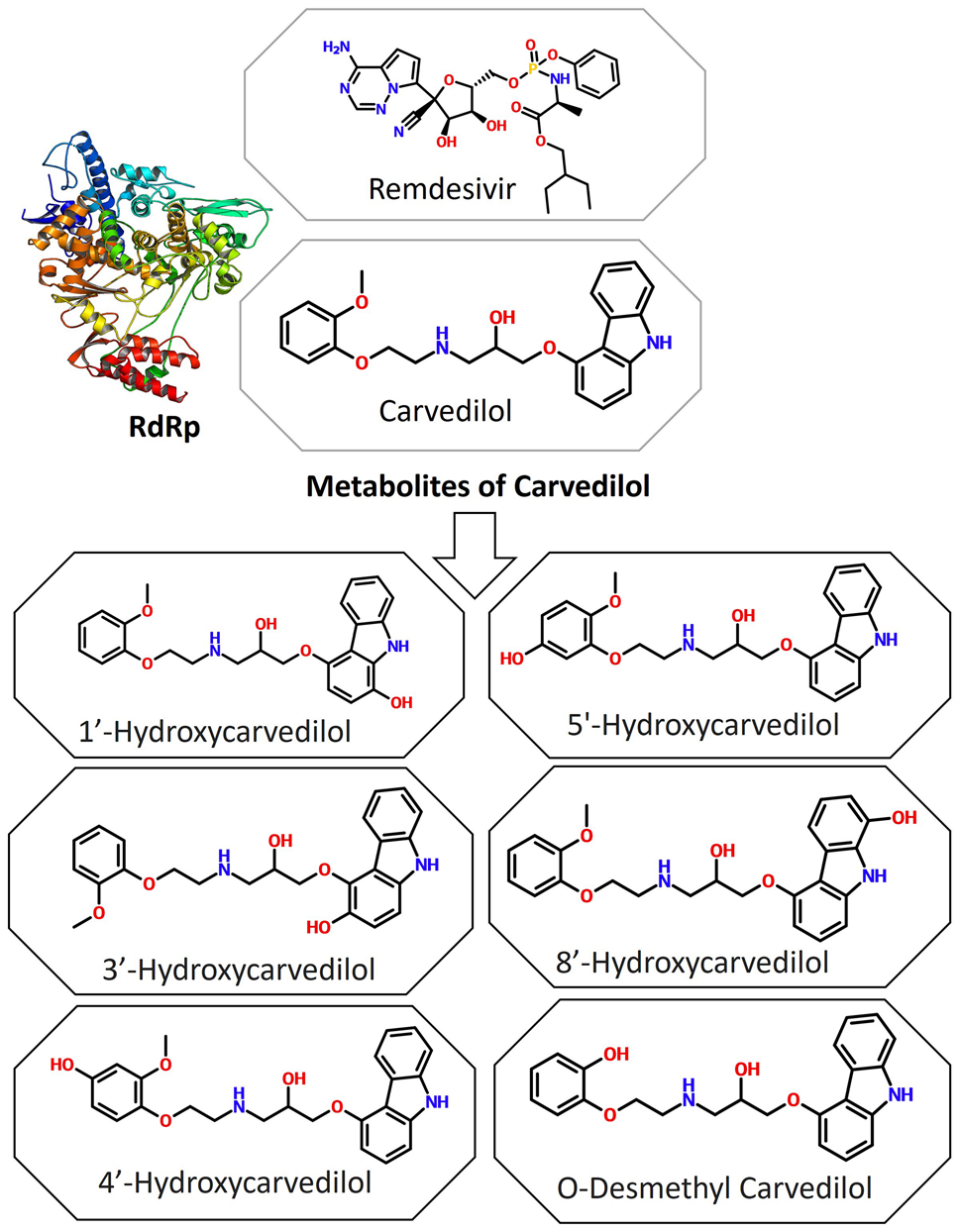
Secondary molecular structures of carvedilol and its metabolites and tertiary structure of RdRp of SARS-CoV-2. Remdesivir is used as a reference for binding affinity.

Considering in vivo metabolites of carvedilol also could bind with viral proteins, we then performed the molecular docking between viral proteins of interest and 6 metabolites of carvedilol^37^, including 1-hydroxyl carvedilol, 3-hydroxyl carvedilol, 4-hydroxyl carvedilol, 5-hydroxyl carvedilol, 8-hydroxyl carvedilol, and O-desmethyl carvedilol (Fig. 2). The docking scores of each metabolite with protein RdRp of SARS-CoV-2 were separately analyzed and shown in a Table 1. The binding affinity scores of each carvedilol metabolite with RdRp were − 9.8 kcal/mol (1-hydroxyl carvedilol), − 9.7 kcal/mol (3-hydroxyl carvedilol), − 9.8 kcal/mol (4-hydroxyl carvedilol), − 9.7 kcal/mol (5-hydroxyl carvedilol), − 10.0 kcal/mol (8-hydroxyl carvedilol), and − 10.1 kcal/mol (O-desmethyl carvedilol), respectively.

In addition to docking with the viral protein RdRp, other viral proteins of interest were also used for molecular docking with 6 metabolites of carvedilol. The results showed that all 6 metabolites of carvedilol displayed a higher binding affinity with the viral RdRp domain compared to their binding affinity with each of the other protein domains (Table 1). Overall, RdRp has the highest binding affinity with carvedilol and its metabolites, suggesting that RdRp is the most potent druggable target for carvedilol and its metabolites. In addition, the properties of carvedilol and its metabolites binding with multiple viral proteins suggest a possibility of exploring antiviral drugs using dual or multiple targets of interest.

### Analysis of the key residues contributing to the interaction of carvedilol and its metabolites with drugable target protein RdRp

**Figure 3:**
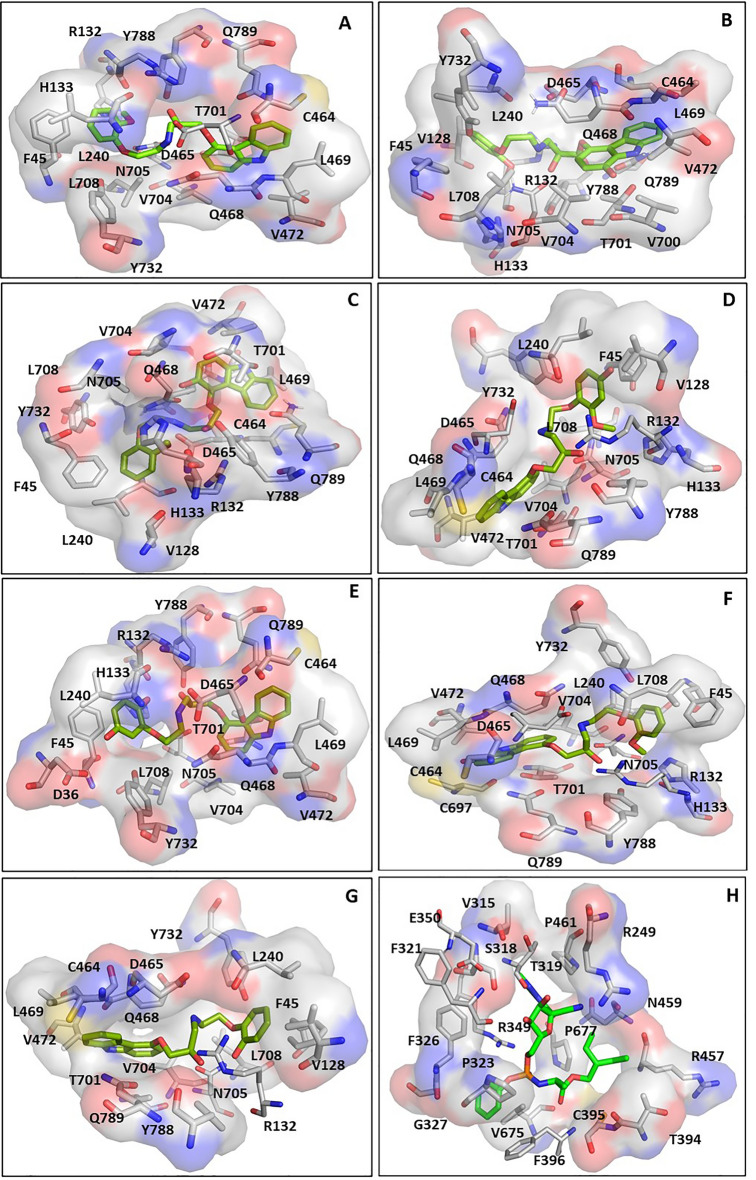
The binding sites of RdRp in the complexes with carvedilol and its metabolites. Remdesivir was applied as a control. (**A**) The binding site of RdRp with carvedilol. (**B**) The binding site of RdRp with 1-hydroxyl carvedilol. (**C**) The binding site of RdRp with 3-hydroxyl carvedilol. (**D**) The binding site of RdRp with 4-hydroxyl carvedilol. (**E**) The binding site of RdRp with 5-hydroxyl carvedilol. (**F**) The binding site of RdRp with 8-hydroxyl carvedilol. (**G**) The binding site of RdRp with O-desmethyl carvedilol. (**H**) The binding site of RdRp with remdesivir.

Given the higher binding affinity of carvedilol and its metabolites with RdRp compared to other virus proteins, we then focused on the analysis of the binding features or properties of carvedilol and its metabolites in complex with RdRp. The key amino acids contributing to the interaction of RdRp with carvedilol and its metabolites were analyzed and shown in a figure (Fig. 3) and listed in a table (Table 2). The results showed that the binding pocket position for carvedilol and its metabolites binding with RdRp was the same. Although RdRp interacted with different ligands, including carvedilol and its metabolites, most of these key residues that contribute to the binding complexes were shared. For example, the residues (F45, R132, L240, C464, D465, Q468, L469, V472 T701, V704, N705, L708, Y732, and Y788) contributed to the interaction of all the complexes, including hydrophobic interactions. In addition to the residues shared by different complexes, some residues such as D36, V128, H133, V700, C697, and Q789 served as the key residues only in some of the complexes. The binding pocket position of carvedilol and its metabolites with RdRp was different from the binding pocket position of remdesivir-RdRp. The key residues involved in the remdesivir-RdRp complex were different from the above-mentioned key residues involved in the complex of carvedilol/its metabolites-RdRp, listed in Table 2.

**Table 2:**
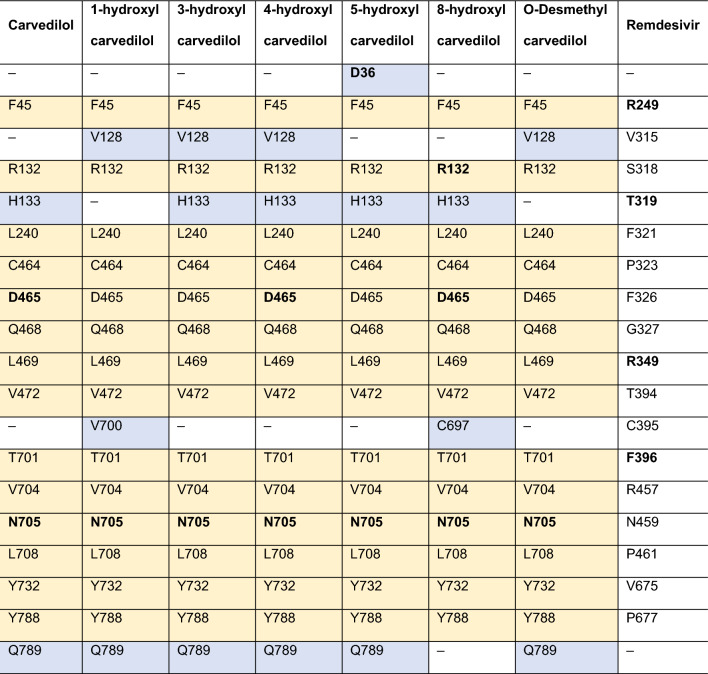
Key residues involved in the interactions between viral protein RdRp and carvedilol and its metabolites.

The interactions between viral protein RdRp and carvedilol (column 1) and its metabolites (columns 2–7) were analyzed, and remdesivir was used as a control (column 8). Residues in bold text contribute to the formation of hydrogen bonds. The yellow highlighted residues stand for the same residues of RdRp that contribute to the ligand–protein interaction between different complexes.

### Analysis of the hydrogen bond and other interactions that contribute to the binding affinity of RdRp with carvedilol and its metabolites

**Figure 4:**
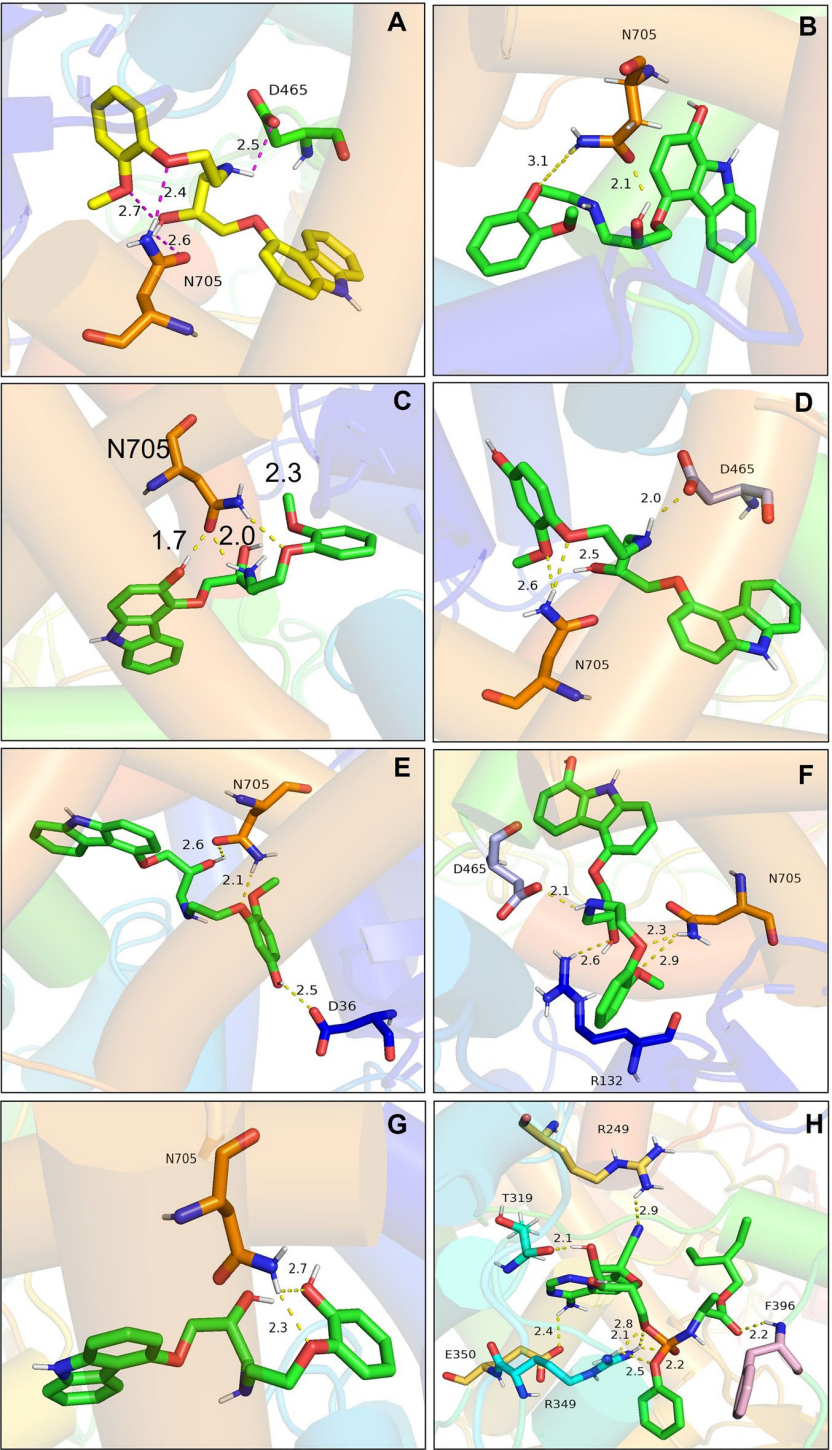
Hydrogen bonds in the binding complexes. The key residues of the RdRp that contribute to the formation of hydrogen bonds in different complexes of RdRp with carvedilol or its metabolites were analyzed. (**A**) carvedilol. (**B**) 1-hydroxyl carvedilol. (**C**) 3-hydroxyl carvedilol. (**D**) 4-hydroxyl carvedilol. (**E**) 5-hydroxyl carvedilol. (**F**) 8-hydroxyl carvedilol. (**G**) O-desmethyl carvedilol. (**H**) The interaction between RdRp and remdesivir was tested as a control. The labeled number is the distance between two atoms that form the hydrogen bond.

The hydrogen bonds contributing to the binding affinity between RdRp and carvedilol and its metabolites were demonstrated in a figure (Fig. 4A–G). In the binding complexes, two or three stable hydrogen bonds were formed between residue N705 of RdRp and carvedilol and its metabolites. In addition to N705, the residues D465 contributed to hydrogen bond formation in the complex of RdRp binding with carvedilol, 4-hydroxyl carvedilol, and 8-hydroxyl carvedilol. Residue D36 formed the hydrogen bond in the complex of RdRp binding with 5-hydroxyl carvedilol, and residue R132 contributed to the hydrogen bond formation in the complex of RdRp binding with 8-hydroxyl carvedilol. In contrast, the residues of RdRp forming hydrogen bonds with remdesivir were different from those in the complexes of RdRp with carvedilol and its metabolites, including R249, T319, R349, E350, and F396 (Fig. 4H). All these residues are highlighted in Table 2, contributing to the protein–ligand interaction.

**Figure 5:**
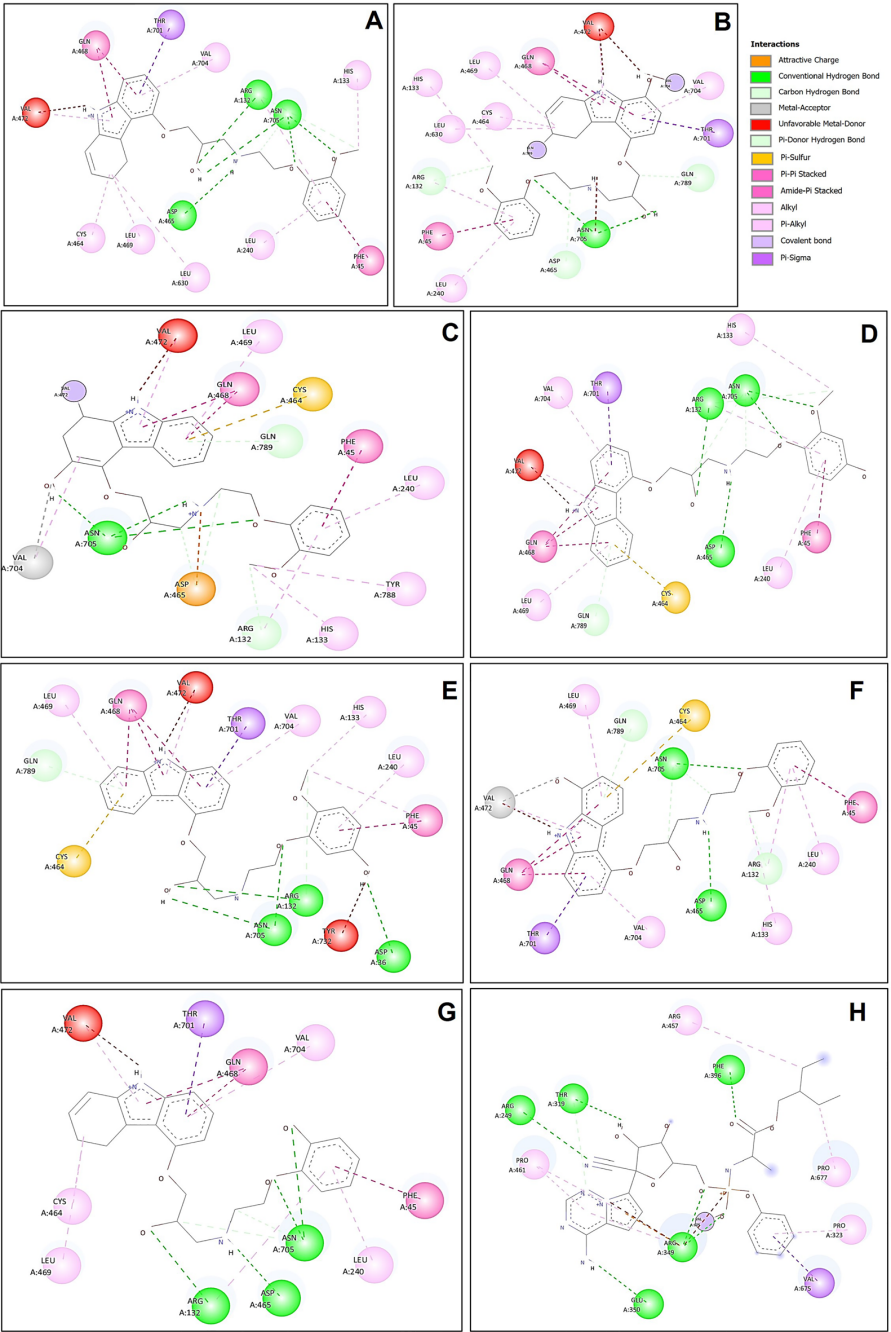
2D graph of all the interactions in the binding sites of RdRp with carvedilolor and its metabolites. (**A**) carvedilol. (**B**) 1-hydroxyl carvedilol. (**C**) 3-hydroxyl carvedilol. (**D**) 4-hydroxyl carvedilol. (**E**) 5-hydroxyl carvedilol. (**F**) 8-hydroxyl carvedilol. (**G**) O-desmethyl carvedilol. (**H**) The interaction between RdRp and remdesivir was also tested and displayed.

In addition to the hydrophobic interaction and hydrogen bonds, the interactions between the ligand and protein also include Van der Waals bonds, Pi–Sigma, Pi–Pi Stack, Pi–Alkyl Pi–Sulfur, Pi–Amide stack, and covalent bonds, contributing to the stability of the protein–ligand complexes (Fig. 5).

### Analysis of the binding pocket of viral RdRp and drugable properties of carvedilol and its metabolites

**Figure 6:**
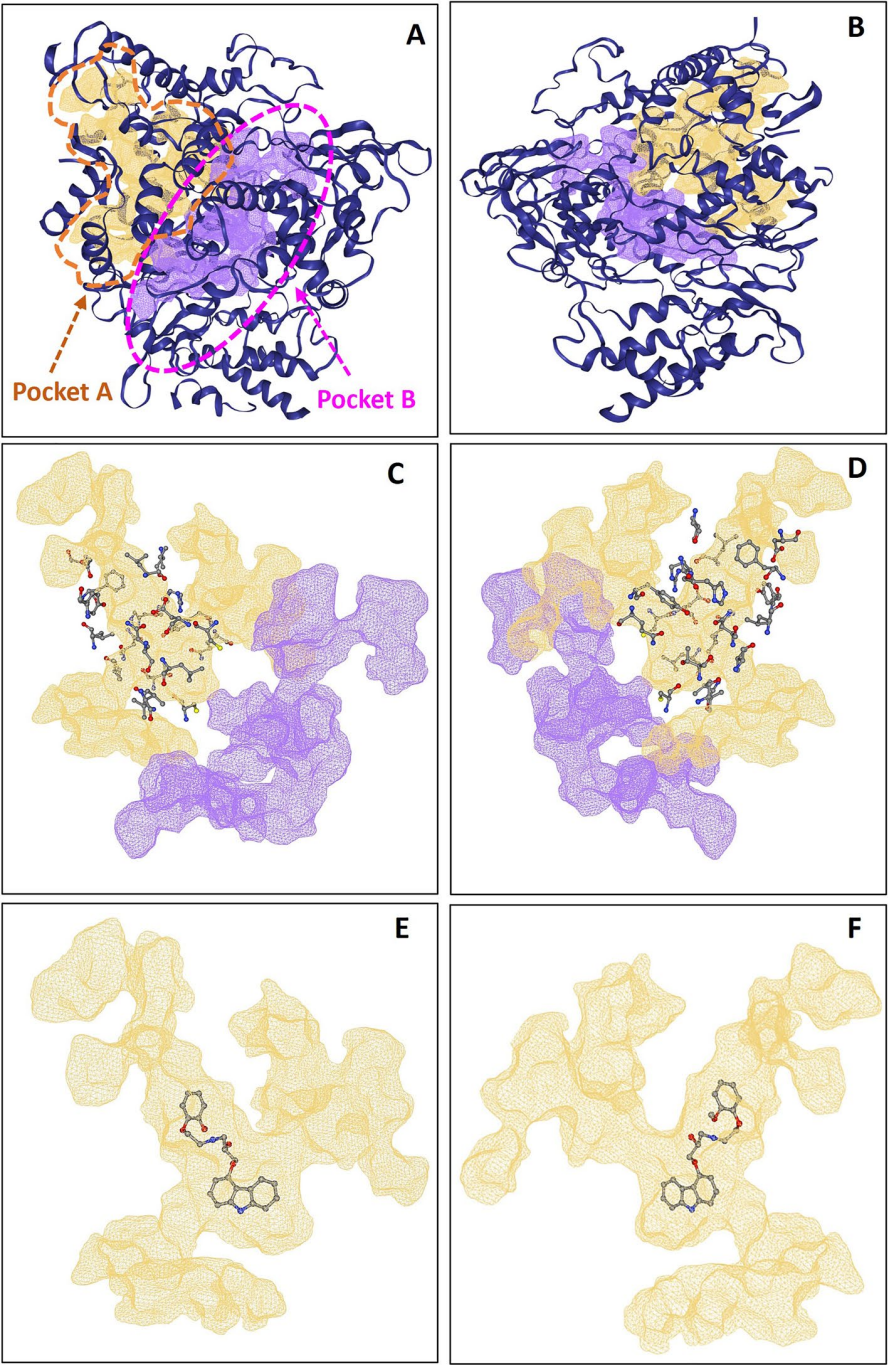
Major binding pockets of RdRp. (**A**) The front side of the two identified major binding pockets (Pocket A in yellow color and Pocket B in purple color). (**B**) The backside of the two identified major binding pockets (Pocket A in yellow color and Pocket B in purple color). The front side (**C**) and backside (**D**) of the two major identified binding pockets. Pocket A (yellow) was surrounded by the key amino acids that contribute to the interaction of the protein–ligand binding between RdRp and carvedilol or its metabolites. The front side (**E**) and backside (**F**) of the binding site of ligand (carvedilol)-RdRp complex used as an example.

To further confirm the availability and druggability of the binding pocket, the computational-based prediction of druggable major pockets of viral RdRp protein was analyzed. Two major druggable pockets were identified with a higher score of druggability 0.81 for both pockets (pocket A and pocket B) (Score range: 0–1) (Fig. 6). The volume [Å^3^] of predicted binding pocket A was 2450.27 and the surface [Å^2^] of predicted binding pocket A was 2553.03. Pocket A was formed by 133 amino acids (Table 3) with functional groups, including 52 hydrogen bond donors, 185 hydrogen bond acceptors, and 53 hydrophobic interactions with a hydrophobicity ratio of 0.18. The amino acid composition of predicted pocket A showed that the apolar amino acid ratio was 0.59, the polar amino acid ratio was 0.31, the positive amino acid ratio was 0.07, and the negative amino acid ratio was 0.04.

**Table 3:** List of the amino acids that form the identified major binding pocket A. 133 amino acids form one of the major pockets (Pocket A) of RdRp (columns 1–7) and the key residues of RdRp in complex with carvedilol and its metabolites (column 8).

133 amino acids that form one of the major pockets (Pocket 1) of RdRp							Key residues
ALA_34_A	VAL_166_A	TYR_217_A	THR_248_A	PRO_620_A	SER_709_A	LEU_775_A	ASP_36_A
PHE_35_A	PRO_169_A	ASP_218_A	ARG_249_A	LYS_621_A	VAL_720_A	VAL_776_A	PHE_45_A
**ASP_36_A**	ILE_171_A	PHE_219_A	TYR_455_A	ARG_624_A	LEU_723_A	ALA_777_A	VAL_128_A
TYR_38_A	LEU_172_A	GLY_220_A	ARG_457_A	ALA_625_A	GLN_724_A	PHE_782_A	ARG_132_A
**PHE_45_A**	VAL_174_A	ASP_221_A	TYR_458_A	PRO_627_A	LEU_727_A	SER_784_A	HIS_133_A
MET_124_A	TYR_175_A	PHE_222_A	ASN_459_A	LEU_630_A	TYR_728_A	VAL_785_A	LEU_240_A
ALA_125_A	ALA_176_A	PRO_232_A	LEU_460_A	MET_633_A	LEU_731_A	LEU_786_A	CYS_464_A
LEU_127_A	LEU_178_A	VAL_233_A	PRO_461_A	ASN_695_A	**TYR_732_A**	TYR_787_A	ASP_465_A
**VAL_128_A**	ARG_181_A	VAL_234_A	THR_462_A	ILE_696_A	ARG_733_A	**TYR_788_A**	GLN_468_A
TYR_129_A	VAL_182_A	SER_236_A	MET_463_A	**CYS_697_A**	PHE_741_A	**GLN_789_A**	LEU_469_A
ALA_130_A	THR_189_A	TYR_237_A	**CYS_464_A**	GLN_698_A	PHE_745_A	ASN_790_A	VAL_472_A
LEU_131_A	PHE_192_A	TYR_238_A	**ASP_465_A**	ALA_699_A	TYR_748_A	ASN_791_A	CYS_697_A
**ARG_132_A**	CYS_193_A	SER_239_A	**GLN_468_A**	**VAL_700_A**	LEU_749_A	VAL_792_A	VAL_700_A
**HIS_133_A**	VAL_204_A	**LEU_240_A**	**LEU_469_A**	**THR_701_A**	HIS_752_A		THR_701_A
PHE_134_A	LEU_205_A	LEU_241_A	PHE_471_A	ALA_702_A	PHE_753_A		VAL_704_A
THR_141_A	THR_206_A	PRO_243_A	**VAL_472_A**	ASN_703_A	MET_755_A		ASN_705_A
LEU_142_A	LEU_207_A	ILE_244_A	VAL_475_A	**VAL_704_A**	ILE_757_A		LEU_708_A
ILE_145_A	ASP_208_A	LEU_245_A	TYR_479_A	**ASN_705_A**	ALA_762_A		TYR_732_A
LEU_146_A	ASN_209_A	THR_246_A	MET_615_A	LEU_707_A	VAL_764_A		TYR_788_A
PHE_165_A	TRP_216_A	LEU_247_A	TRP_617_A	**LEU_708_A**	TYR_770_A		GLN_789_A

Residues in bold are the key residues that contribute to the formation of RdRp in complexes with carvedilol and its metabolites.

The results from our study showed the binding position of carvedilol and its metabolites were located within the major binding pocket A. All the key amino acids that contributed to the interaction of the binding affinity of the protein–ligand complex between the RdRp and carvedilol or its metabolites were included in the 133 amino acids that formed pocket A (Table 3). The size of binding pockets of carvedilol and its metabolites were smaller compared to pocket A, and the predicted druggable major binding pocket of these molecules was exactly located inside of the major binding pocket A. These results suggest that carvedilol and its metabolites have druggable properties against SARS-CoV-2 infection by targeting viral RdRp domain.

### Molecular dynamic simulation to validate the binding of complexes

**Figure 7:**
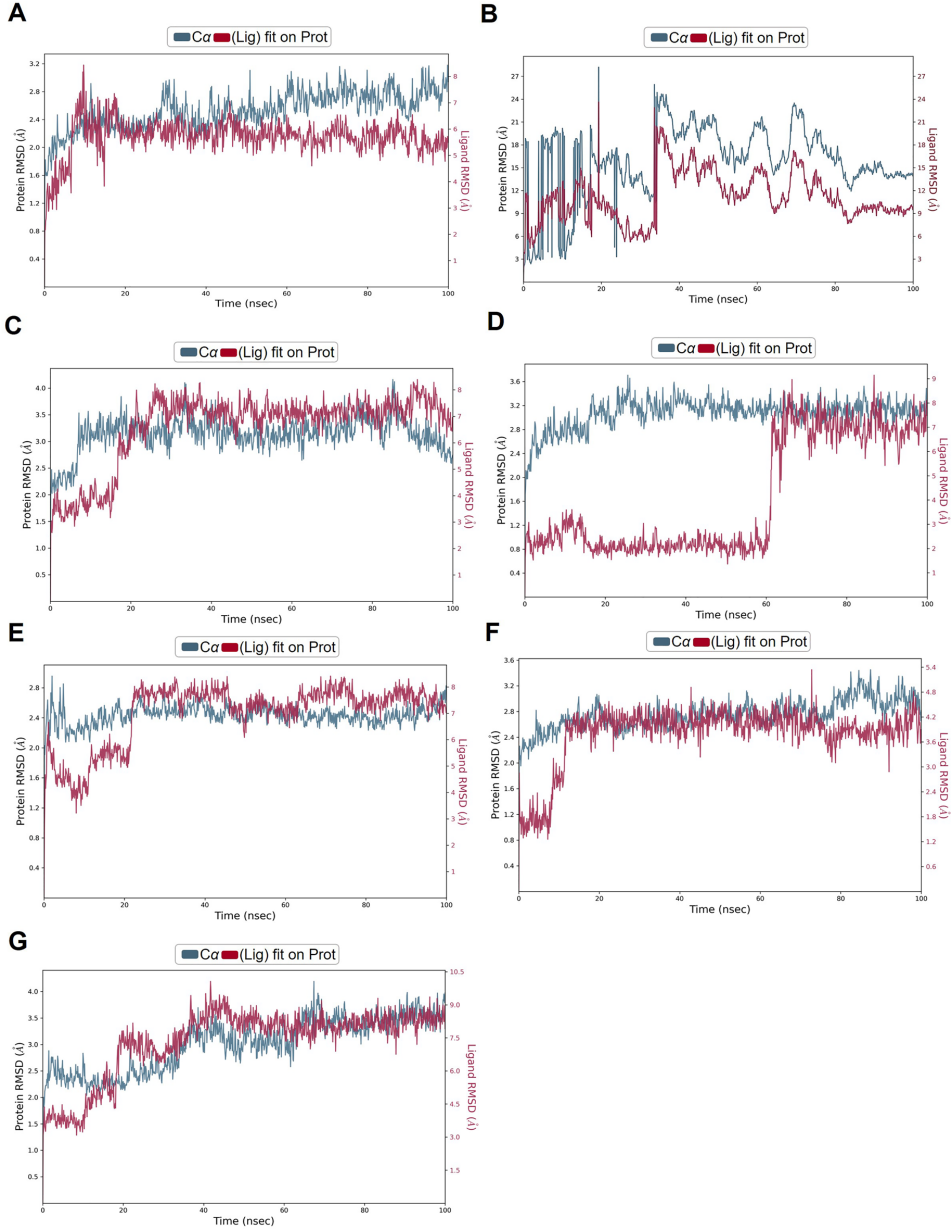
Results of molecular dynamics simulation of RdRp in complex with carvedilol and its metabolites. (**A**) The complex of RdRp (PDB ID: 6XQB) and carvedilol. (**B**) The complex of RdRp and 1-hydroxyl carvedilol. (**C**) The complex of RdRp and 3-hydroxyl carvedilol. (**D**) The complex of RdRp and 4-hydroxyl carvedilol. (**E**) The complex of RdRp and 5-hydroxyl carvedilol. (**F**) The complex of RdRp and 8-hydroxyl carvedilol. (**G**) The complex of RdRp and O-Desmethyl carvedilol.

**Figure 8:**
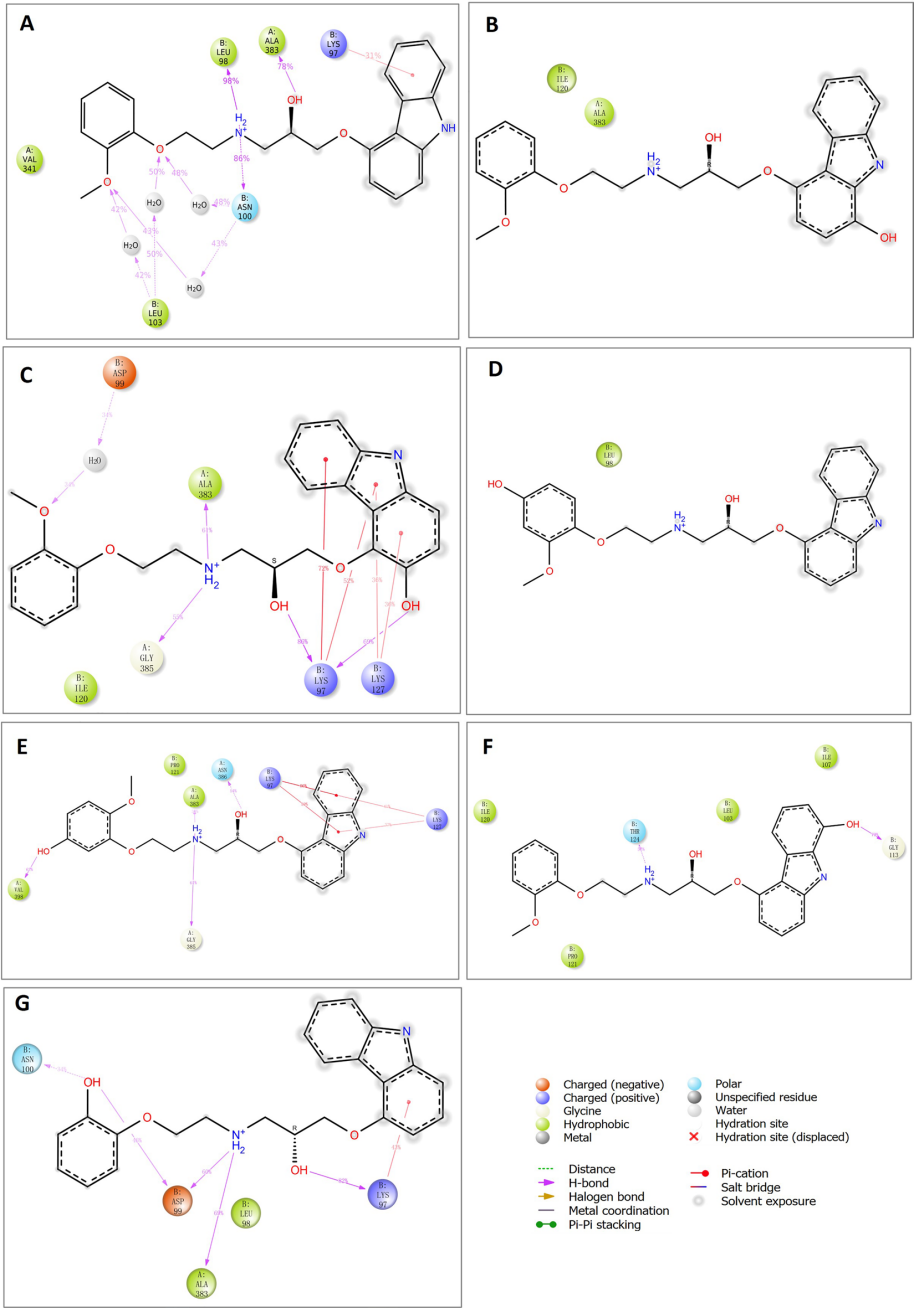
2D schematic diagrams of the ligand atoms interacting with target proteins. The ligand atoms of carvedilol and its metabolites interacting with target protein RdRp (PDB ID: 6XQB) were analyzed, respectively. (**A**) The complex of RdRp and carvedilol. (**B**) The complex of RdRp and 1-hydroxyl carvedilol. (**C**) The complex of RdRp and 3-hydroxyl carvedilol. (**D**) The complex of RdRp and 4-hydroxyl carvedilol. (**E**) The complex of RdRp and 5-hydroxyl carvedilol. (**F**) The complex of RdRp and 8-hydroxyl carvedilol. (**G**) The complex of RdRp and O-desmethyl carvedilol. Labeled numbers stand for the percentages of contributions of atoms for the binding within 100 ns simulation time range.

**Figure 9:**
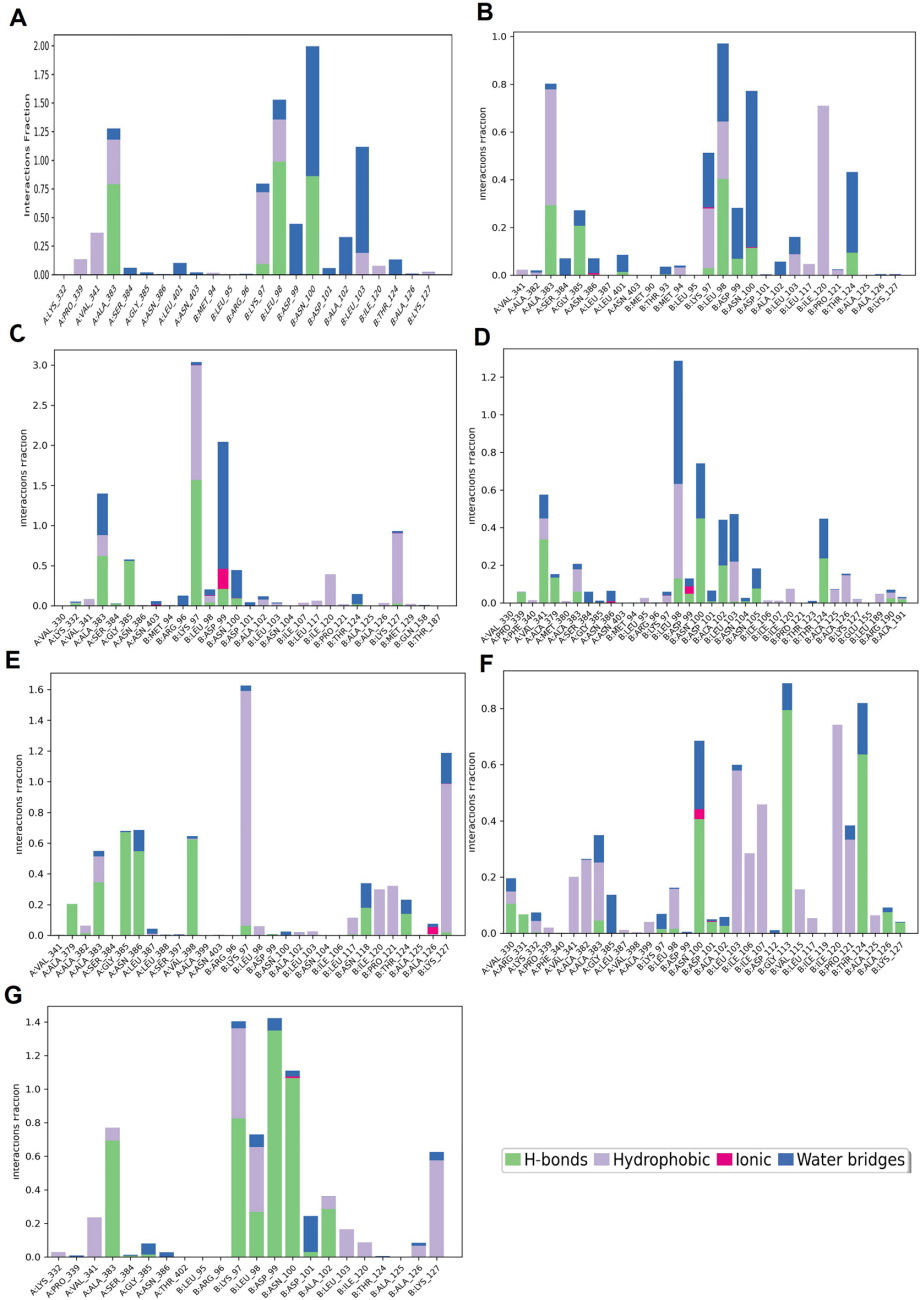
Stacked bar chart plot showing the protein-ligands interactions found during the 100 ns simulation run. The X-axis shows the residues of the protein that contribute to the protein–ligand interaction under the four categories including hydrogen bonds, hydrophobic, ionic, and water bridges. The residues from RdRp NSP12 (displayed as chain A) and amino acids from co-factors NSP8 (displayed as Chain B) (PDB ID: 6XQB). The Y-axis stands for the interaction fraction that describes how long interactions are maintained over the simulation run. An interaction fraction of 1.0 suggests that this interaction is maintained in the simulation 100% of the time. If the value is > 1.0, this means that the protein residue is involved in multiple interaction contacts.

**Figure 10:**
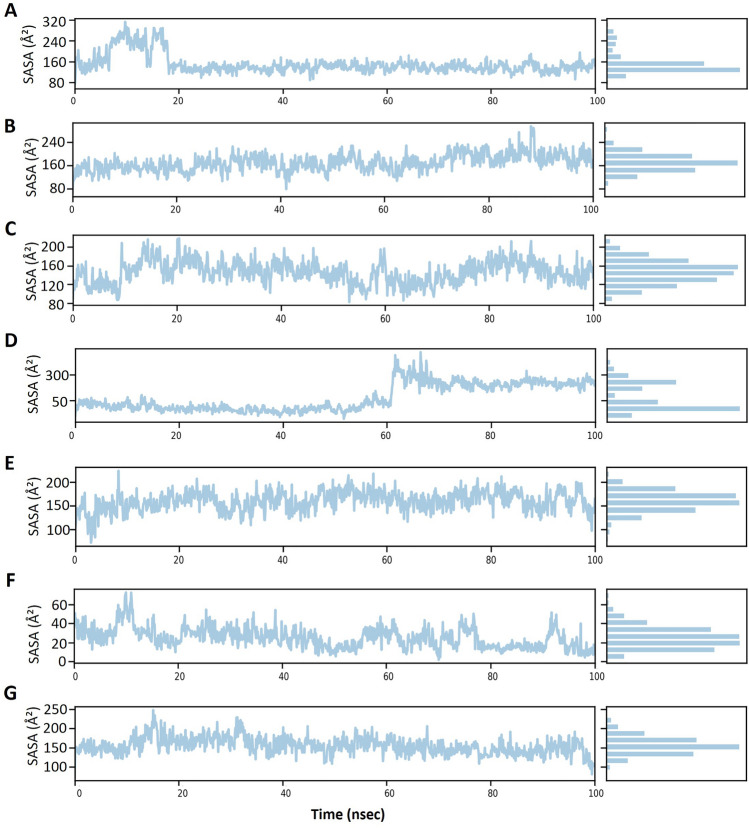
Solvent accessible surface area (SASA) results from the molecular dynamic simulation of the ligand–protein complexes. The interactions include carvedilol and its metabolites interacting with the target protein RdRp (PDB ID: 6XQB), respectively. (**A**) The complex of RdRp and carvedilol. (**B**) The complex of RdRp and 1-hydroxyl carvedilol. (**C**) The complex of RdRp and 3-hydroxyl carvedilol. (**D**) The complex of RdRp and 4-hydroxyl carvedilol. (**E**) The complex of RdRp and 5-hydroxyl carvedilol. (**F**) The complex of RdRp and 8-hydroxyl carvedilol. (**G**) The complex of RdRp and O-desmethyl carvedilol. The simulation time is 100 ns, SPC (simple point charge) water model as solvent for simulation was used.

To further test the binding stability between carvedilol and target proteins, we chose the most conserved viral protein RdRp comprising a catalytic subunit NSP12 and two acessary subunits NSP7 and NSP8, also known as a target of FDA-approved drug remdesivir, to conduct the molecular simulation. Within the simulated time range of 100 ns, except for the RdRp-1-hydroxyl carvedilol complex (Fig. 7B), all the other tested complexes reached a relatively stable stage (Fig. 7A and C–G). The root mean square fluctuation (RMSF)^38^ results of proteins and ligands were further analyzed to test the fluctuation of atoms around the average position (Supplementary Fig. 1). These tested complexes include carvedilol-RdRp and carvedilol metabolite-RdRp complexes. The interacting features and properties of molecules with target proteins were characterized and analyzed for each complex, respectively. For instance, these interactions included hydrogen bound, hydrophobicity, charge distribution, polarization, Pi-cation, etc. (Fig. 8). The interacting residues and fractions that contributed to the interaction of hydrogen bonds, hydrophobic, ionic, and water bridges were analyzed (Fig. 9). These residues included the amino acids from RdRp NSP12 (displayed in chain A, Fig. 9) and amino acids from co-factors NSP8 (displayed in Chain B, Fig. 9). The solvent accessible surface area (SASA) measures the surface area of a molecule that is accessible to a solvent, which is a valuable metric for protein–ligand interactions. The fluctuation of SASA for carvedilol and its metabolites in complex with RdRp was displayed in the result of SASA profile generated within 100 ns of dynamic simulation (Fig. 10).

AMDET analysis results showed that all the tested molecules meet the Lipinski drug-likeness criteria and were accepted by the criteria applied by Pfizer for drug discovery screening. The drug-likeness results also met the criteria of Egan (Pharmacia) filter and Muegge (Bayer) filter for drug screening (Table 4).

**Table 4:** Pharmacokinetic properties ADMET analyses of absorption, distribution, excretion, metabolism, toxicity, and drug-likeness assessment.

ADMET	Carvedilol	1-hydroxyl carvedilol	3-hydroxyl carvedilol	4-hydroxyl carvedilol	5-hydroxyl carvedilol	8-hydroxyl carvedilol	O-desmethyl carvedilol
Physicochemical Properties							
Molecular Weight	406.47	422.47	422.47	422.47	422.47	422.47	392.45
H-bond acceptors	5	6	6	6	6	6	5
H-bond donors	3	4	4	4	4	4	4
Number of rings	4	4	4	4	4	4	4
Number of carbons	> 4	> 4	> 4	> 4	> 4	> 4	> 4
Rotatable bonds	10	10	10	10	10	10	9
Heteroatoms	6	7	7	7	7	7	6
TPSA	75.74	95.97	95.97	95.97	95.97	95.97	86.74
Lipophilicity							
iLOGP	3.45	2.15	3.2	2.31	3.41	3.08	2.48
XLOGP3	4.37	4.01	4.01	4.01	4.01	4.01	4.1
WLOGP	3.74	3.44	3.44	3.44	3.44	3.44	3.44
MLOGP	1.96	1.43	1.43	1.43	1.43	1.43	1.75
Consensus Log P	3.61	3.02	3.23	3.05	3.27	3.21	3.15
Pharmacokinetics							
GI absorption	High	High	High	High	High	High	High
BBB permeant	Yes	No	No	No	No	No	No
Pgp substrate	Yes	Yes	Yes	Yes	Yes	Yes	Yes
CYP1A2 inhibitor	Yes	Yes	Yes	Yes	Yes	Yes	Yes
CYP2C19 inhibitor	Yes	Yes	Yes	Yes	Yes	Yes	Yes
CYP2C9 inhibitor	Yes	Yes	Yes	Yes	Yes	Yes	Yes
CYP2D6 inhibitor	Yes	Yes	Yes	Yes	Yes	Yes	Yes
CYP3A4 inhibitor	Yes	Yes	Yes	Yes	Yes	Yes	Yes
log Kp (cm/s)	− 5.68	− 6.03	− 6.03	− 6.03	− 6.03	− 6.03	− 5.78
Drug-likeness							
Lipinski (Pfizer)	Yes	Yes	Yes	Yes	Yes	Yes	Yes
Egan (Pharmacia)	Yes	Yes	Yes	Yes	Yes	Yes	Yes
Muegge (Bayer)	Yes	Yes	Yes	Yes	Yes	Yes	Yes
Bioavailability	0.55	0.55	0.55	0.55	0.55	0.55	0.55

Criteria for Lipinski (Pfizer) filter: molecular weight ≤ 500, H-bond acceptors ≤ 10, H-bond donors ≤ 5, MLOGP ≤ 4.15; Criteria for Egan (Pharmacia) filter: WLOGP ≤ 5.88, TPSA ≤ 131.6; Criteria for Muegge (Bayer) filter: 200 ≤ molecular weight ≤ 600, -2 ≤ XLOGP ≤ 5, TPSA ≤ 150, number of rings ≤ 7, number of carbon > 4, Rotatable bonds ≤ 15, Heteroatoms > 1, H-bond acceptors ≤ 10, H-bond donors ≤ 5.

TPSA: Topological polar surface area; iLOGP, XLOGP3, WLOGP, MLOGP, and Silicos-IT Log P are five methods used to calculate the n-octanol/water partition coefficient (log Po/w); Consensus Log P: coefficient average value calculated by the aforementioned five methods; GI absorption: Gastrointestinal absorption; BBB permeant: blood–brain barrier permeant; Pgp substrate: P-glycoprotein substrate; CYP1A2: Cytochrome P450 1A2; CYP2C19: Cytochrome P450 2C19; CYP2C9: Cytochrome P450 2C9; CYP2D6: Cytochrome P450 2D6; CYP3A4: Cytochrome P450 3A4; log Kp (cm/s): Skin permeability coefficient.

## Discussion

Repurposing the FDA-approved drug for medical use to treat diseases can provide several benefits from different perspectives compared to de-novo drug discovery, such as the time-saving process, low cost for drug development, and reduced load of safety testing for applications^39^. For example, using molecular docking and dynamics simulation studies to screen chemical compounds from natural products against the receptor-binding domain of SARS-CoV-2 provides many drug candidates for further validation in wet labs^40^.

Molecular docking is an essential computational approach in drug discovery, allowing the exploration of potential leading drugs or compounds, identifying suitable therapeutic drugs, and accelerating drug design and development^41–44^. Computer-aided drug discovery is also a powerful tool to facilitate the mechanistic investigation of drugs and their targets, such as the binding affinity, binding mode, and essential residues that contribute to the binding interaction and stability of the complex between molecules and their target proteins^45–50^. Most importantly, the comprehensive and rational docking experiments applied in the computational approach should be based on the evidence of clinical observation and a deep understanding of the properties of the molecules and diseases, which is critical for harnessing the efficacy of drug discovery using computational methodologies and approaches^51–53^.

Results of clinical observation, network analysis, and in vitro studies have demonstrated that there are beneficial effects of the usage of carvedilol against SARS-CoV-2 infection. In this study, we explored the potential usage of carvedilol and its metabolites as re-purposed drugs to fight against SARS-CoV-2 infection using computer-aided mechanistic analysis. Our results have demonstrated that both carvedilol and its metabolites showed the properties to bind with multiple viral proteins (Table 1). The binding affinity of carvedilol/metabolites with RdRp showed the highest score compared to other tested viral proteins. RdRp can serve as the most promising druggable site for both carvedilol and its metabolites (Fig. 6).

Molecular dynamics simulation is a powerful computational method and invaluable tool in drug discovery, aiming to simulate the actual physical system and process^54,55^. Computing power also allows the characterization and analysis of ligand–protein interaction, which provides important information for understanding the biological and molecular mechanisms of small molecules and target proteins. The analysis results can also provide knowledge-based insight for further modification and optimization of druggable compounds. The results from this study suggest that there is a relatively stable binding complex formed between viral protein RdRp and carvedilol, as well as its metabolites with RdRp (Fig. 7). These results indicate repurposing carvedilol as a potential drug against COVID-19 infection exerts further investigation in research studies.

Therefore, the computational approach is a valuable and promising strategy to perform drug repurposing. Especially, in the pandemic area, computational-aided drug discovery could provide an unreplaceable method to accelerate the pace of drug discovery. During the COVID-19 pandemic, a lot of investigation and progress has been made using this approach^56–58^. Most importantly, the mutation and variant of interest could also be deeply investigated using the computational repurposing method^59,60^. In the current study, we incorporated the drug action mechanism of the FDA-approved drug carvedilol and clinical observation using a molecular docking approach to explore the potential of repurposing carvedilol as an anti-viral agent against SARS-CoV-2 infection. The molecular dynamic simulation was used to further confirm the findings. As demonstrated by the dynamic results, within the 100 ns dynamic simulation, the complexes could reach a relatively stable stage, except for the complex of RdRp and 1-hydroxyl carvedilol shown in Figure (Fig. 7B).The results of ADMET analysis results of the candidate molecules including carvedilol and its metabolites further demonstrated the feasibility of using those molecules as anti-viral compounds for future investigation.

For future perspectives, both in vitro and in vivo studies are needed to verify the treatment efficacy of those molecules. The exploration and optimization of the dosages are also required for the application in SARS-CoV-2 infection treatment. Meanwhile, reducing or minimizing the side effects is important given its functionality as a blood pressure modulator. The dual or multiple drug targets need to be further investigated to gain a synergistic effect against viral infection, considering the multiple druggable binding sites and several potential target proteins in the virus^61^. Moreover, given the modulatory properties of carvedilol on the levels of IL-6, the immune modulators may be applied as a synergistic treatment to improve the host immune system to result in better efficacy against viral infection^62,63^.

Drug repurposing is an alternative approach for developing new disease treatments, which has multiple benefits, such as low cost for drug development and risk assessment. However, the original effects of a drug should also be considered when applying it as a repositioning drug. Carvedilol can treat high blood pressure; therefore, it might not be applicable to use it as an anti-viral drug in subjects with low blood pressure or hypotension. In two conditions, repositioning drugs may be more applicable, including (1) incorporating the established function and repositioning function together to enhance treatment efficacy in subjects with both diseases and (2) shifting the original function to a newly proposed therapy for disease treatment. The side effects derived from the drug's original function and its application dosage of a repurposed drug for COVID-19 treatment should be evaluated and measured, while they are applied in patients with only SARS-CoV-2 infection. Newly merged studies suggest the possibility of SARS-CoV-2 infection can cause high blood pressure or hypertension^64^. Thus, the blood control agent carvedilol will confer a beneficial effect for controlling both blood pressure and viral infection. However, it may not be applicable for patients with only viral infections, as reducing the blood pressure function of carvedilol is a side effect. Overall, well-designed studies are needed to further assess the functions of the repurposed drugs. Modification and optimization of these agents may be necessary before the clinical trials.

In conclusion, based on computational analysis and molecular dynamics simulation, this study provides promising insight for further investigation of using carvedilol and its metabolites as repurposed drugs to fight against SARS-CoV-2 infection.

## Materials and methods

### Protein preparation

The crystal structure protein models used for docking in this study were originally obtained from the Protein Data Bank (www.rcsb.org): including the RNA-directed RNA polymerase, RdRp (PDB ID:6XQB) with a resolution of 3.40 Å, and the Chain A used for this study; the crystal structure of COVID-19 main protease 3CL/Mpro (PDB ID:6LU7, resolution of 2.16 Å, Chain A); was the crystal structure of NSP13 helicase (PDB ID:7CYQ, resolution of 2.83 Å, Chain E); non-structural protein 2 (NSP2) (PDB ID: 7MSX, resolution of 3.15 Å); papain-like protease NSP3/PLpro (PDB ID:7BF6, resolution 2.15 Å, chain A); SARS-CoV-2 spike protein S1 receptor-binding domain RBD (PDB ID: 6M0J, resolution of 2.45 Å, ligand removed); the crystal structure of human angiotensin-converting enzyme 2 (ACE2, PDB ID: 6M0J, resolution of 2.45 Å; and the crystal structure of the RBD-ACE2 complex (PDB ID: 6M0J, resolution of 2.45 Å). All these proteins were prepared for docking such as adding hydrogen, removal of water, elimination of all non-standard residues, adding Gasteiger charges, and removal of ligands if necessary.

### Preparation of small molecules

Carvedilol (Compound CID: 2585), 1-hydroxyl carvedilol (Compound CID: 182523), 3-hydroxyl carvedilol (Compound CID: 127014), 4-hydroxyl carvedilol (Compound CID: 4572774), 5-hydroxyl carvedilol (Compound CID: 4181439), 8-hydroxyl carvedilol (Compound CID: 9979639), O-desmethyl carvedilol (Compound CID: 155763), remdesivir (Compound CID: 121304016) were obtained from the https://pubchem.ncbi.nlm.nih.gov/database. All the molecules were ensured with the hydrogen added and prepared for docking.

### Molecular docking

Molecular modeling approach is a useful method for drug discovery^65^. Molecular docking was performed using an online server CB-Dock that adopted the docking and calculation algorithms based on the Auto Dock Vina (version 1.1.2). The blind docking was performed without defining the grid box to ensure that the best binding site with the highest binding affinity was identified. The parameter of identified best binding cavities between carvedilol and proteins of interest were listed in detail: 3CL/Mpro (Volume = 688; Center: x = − 23.771; y = 1.402; z = 56.178; Size: x = 26, y = 26, z = 26). NSP13 Helicase (Volume = 377; Center: x = 241.975; y = 170.098; z = 173.922; Size: x = 26, y = 26, z = 26). NSP2 (Volume = 561; Center: x = 126.469; y = 114.941; z = 117.846; Size: x = 21, y = 21, z = 21). PLpro (Volume = 570; Center: x = 22.645; y = − 3.141; z = 3.537; Size: x = 26, y = 26, z = 26). RBD (Volume = 94; Center: x = − 20.253; y = 26.578; z = 35.025; Size: x = 26, y = 26, z = 26). ACE2 (Volume = 377; Center: x = − 30.131; y = 32.105; z = − 21.789; Size: x = 26, y = 26, z = 26). RBD-ACE2 complex (Volume = 706; Center: x = − 30.503; y = 17.837; z = − 2.023; Size: x = 26, y = 26, z = 26). The best model with the highest binding affinity was identified for RdRp in complex with carvedilol and its metabolites (Volume = 783; Center: x = 101.643, y = 95.028, z = 80.186; Size: x = 26, y = 26, z = 26). Remdesivir Volume = 4738; Center: x = 106.442, y = 107.143, z = 103.191; Size: x = 25, y = 31, z = 31)^66–68^.

Docking complexes used for molecular dynamics simulation were prepared using crystal structure from PDB ID: 6XQB with all chains kept and containing RdRp (NSP12) in complex with its co-factors NSP7 and NSP8.

### Binding pocket identification

RNA-directed RNA polymerase, RdRp, the major binding pockets identification was performed using prepared protein obtained from the crystal structure from Protein Data Bank (www.rcsb.org) and online server bioinformatics analysis tool https://proteins.plus/^69–71^.

### Analysis of interaction on binding sites

The analysis and visualization of interaction on binding sites and 2D interaction graphs were generated using LigPlus (V.2.2.4)^72^, PyMol Molecular Graphics System (Version 2.0 Schrödinger, LLC) software, and Discover Studio Visualizer (2021) software.

### Molecular dynamic simulation

To further confirm the stability of the molecules in complex with RdRp, the molecular dynamics (MD) simulation was carried out up to 100 ns by applying the Desmond package (the force field OPLS2005 (Schrödinger, LLC, New York, NY, 2015). The calculation of effective evaluation parameters was conducted, such as the root mean square deviation (RMSD) and the root mean square fluctuation (RMSF). RMSD referred to the overall spatial difference between the reference protein compared to the protein that was docked with ligand, which was used to measure the conformational stability of the complex, while the RMSF referred to the individual residue fluctuation (flexibility) during the simulation, which was also used to calculate and measure the stability of the docked complex. The intermolecular interactions of protein–ligand were also calculated to examine the stability and strength of the docked complex over the simulation period. The MD simulation was carried out under thermodynamic conditions (applied volume, density, pressure, and temperature) with the annealed and equilibrated complete system^73,74^.

### Pharmacokinetic properties ADMET and druglikeness evaluation

The platform SWISSADME was used for systematic analysis of the absorption, distribution, excretion, metabolism, and toxicity (ADMET), as well as the evaluation of drug-likeness for the current study^75^.

### Supplementary Information

Supplementary Figure 1.

## Data Availability

All the data supporting reported results can be found publicly and presented in the current study.
