# Supplementary material for: In silico drug repurposing carvedilol and its metabolites against SARS-CoV-2 infection using molecular docking and molecular dynamic simulation approaches
Source: Sci Rep. 2023 Dec 4;13:21404. doi: 10.1038/s41598-023-48398-6 (PMC10696093; doi:10.1038/s41598-023-48398-6)
Supplement: Supplementary file 1 — Supplementary Figure 1. [file 41598_2023_48398_MOESM1_ESM.pdf]

In silico drug repurposing carvedilol and its metabolites against SARS-CoV-2 infection using molecular docking and molecular dynamic simulation approaches

Chunye Zhang[1], Jiazheng Liu[2], Yuxiang Sui[3], Shuai Liu[4], and Ming Yang[5,6,*]

[1]Christopher S. Bond Life Sciences Center, University of Missouri, Columbia, Missouri 65212, USA;

[2]State Key Laboratory of Quality Research in Chinese Medicine, Macau Institute for Applied Research in Medicine and Health, Macau University of Science and Technology, Taipa, Macau 999078, China;

[3]School of Life Science, Shanxi Normal University, Linfen, Shanxi province 041004, China;

[4]The First Affiliated Hospital, Zhejiang University, Hangzhou, Zhejiang province 310006, China;

[5]Department of Surgery, University of Missouri, Columbia, Missouri 65212, USA;

[6]NextGen Precision Health Institution, University of Missouri, Columbia, Missouri 65212, USA;

*****Correspondence: yangmin@health.missouri.edu

Supplementary Figure 1 and legend:

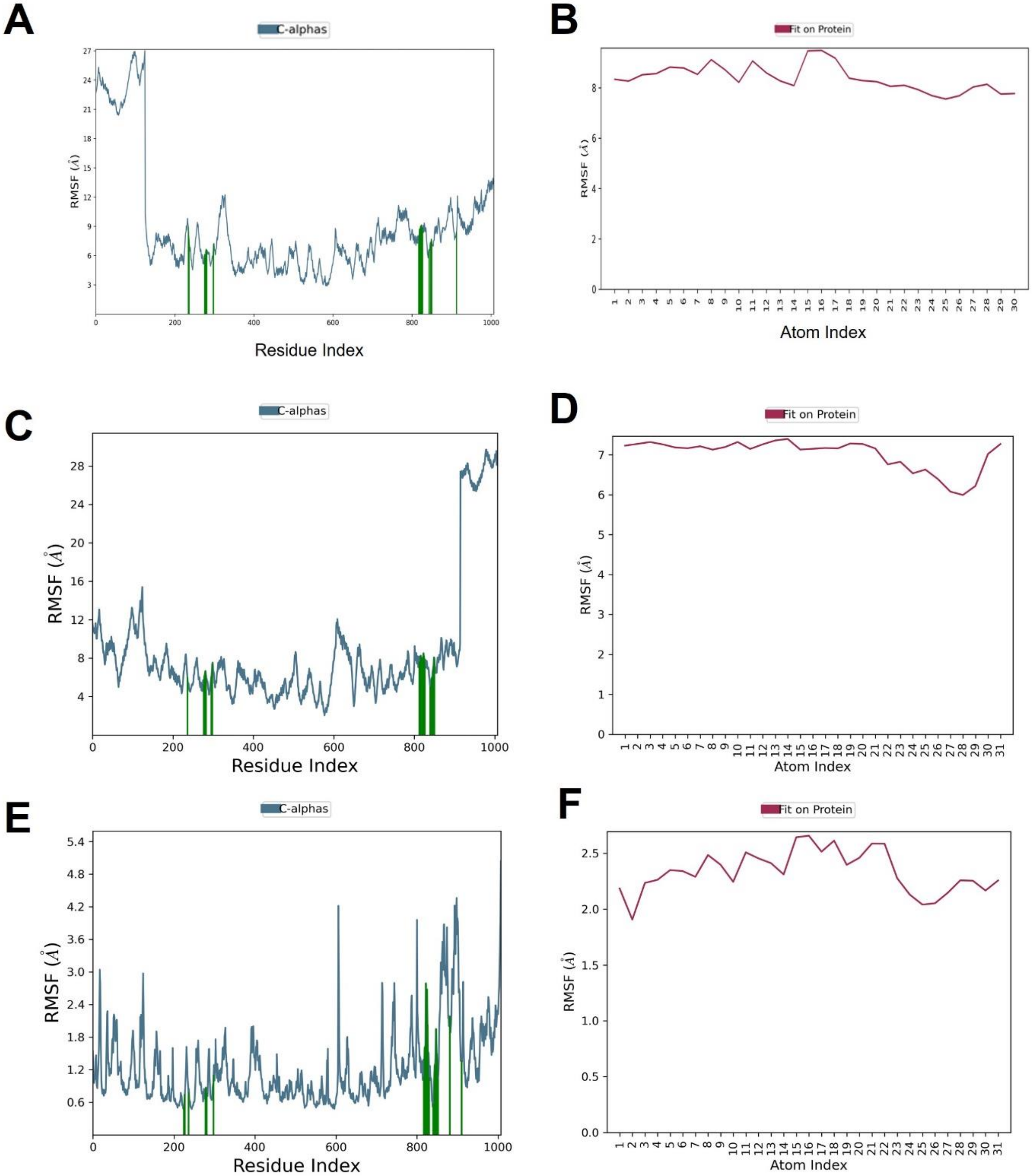

A
C-alphas
RMSF (Å)
Residue Index
B
Fit on Protein
RMSF (Å)
Atom Index
C
C-alphas
RMSF (Å)
Residue Index
D
Fit on Protein
RMSF (Å)
Atom Index
E
C-alphas
RMSF (Å)
Residue Index
F
Fit on Protein
RMSF (Å)
Atom Index

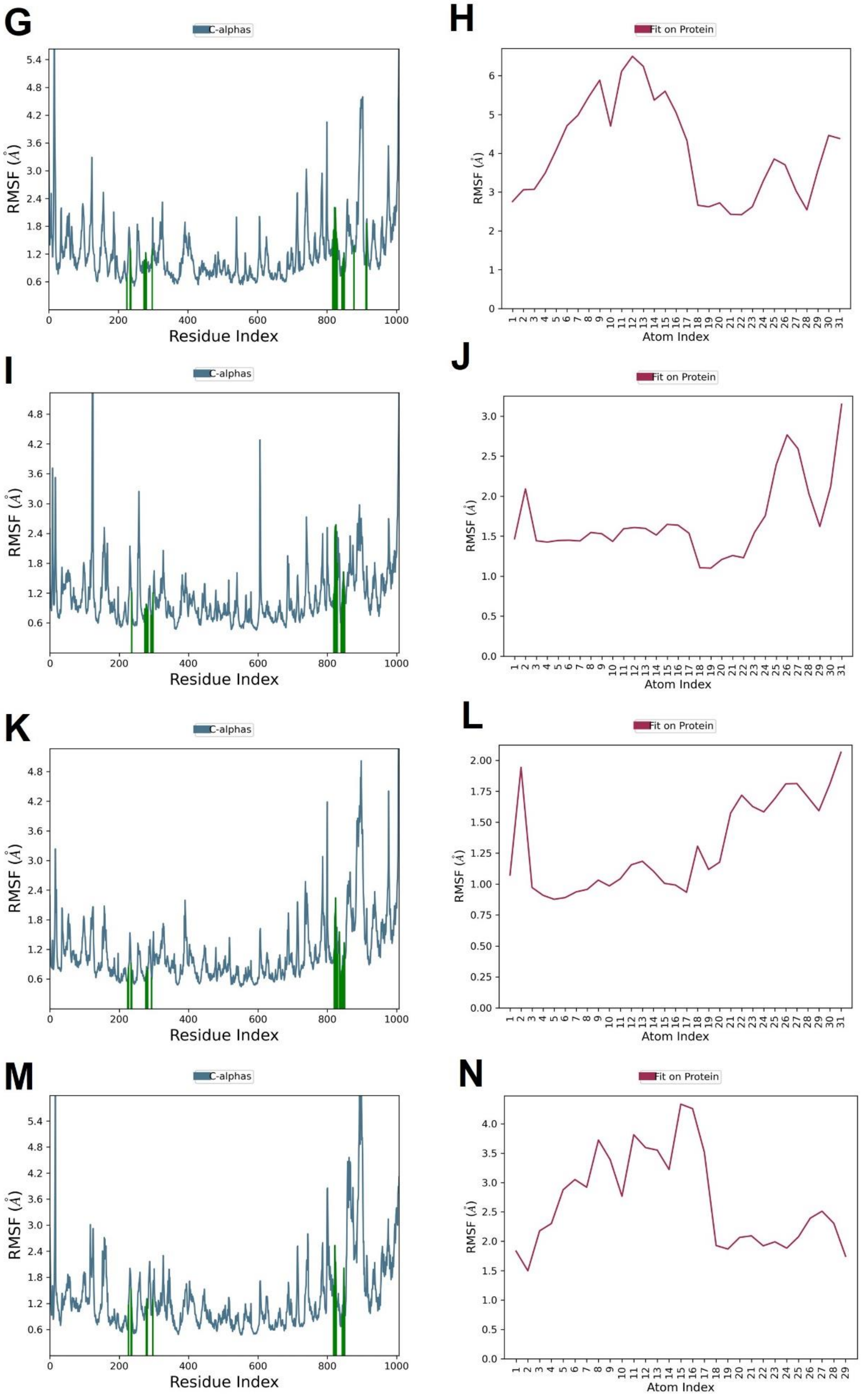
G
C-alphas
RMSF (Å)
Residue Index
H
Fit on Protein
RMSF (Å)
Atom Index
I
C-alphas
RMSF (Å)
Residue Index
J
Fit on Protein
RMSF (Å)
Atom Index
K
C-alphas
RMSF (Å)
Residue Index
L
Fit on Protein
RMSF (Å)
Atom Index
M
C-alphas
RMSF (Å)
Residue Index
N
Fit on Protein
RMSF (Å)
Atom Index

Supplementary Figure 1. The Root Mean Square Fluctuation (RMSF) of protein and ligands. (A) RMSF of RdRp protein of SARS-CoV-2 with (B) RMSF of ligand Carvedilol. (C) RMSF of RdRp protein with (D) RMSF of ligand 1-hydroxyl carvedilol. (E) RMSF of RdRp protein with (F) RMSF of ligand 3-hydroxyl carvedilol. (G) RMSF of RdRp protein with (H) RMSF of ligand 4-hydroxyl carvedilol. (I) RMSF of RdRp protein with (J) RMSF of ligand 5-hydroxyl carvedilol. (K) RMSF of RdRp protein with (L) RMSF of ligand 8-hydroxyl carvedilol. (M) RMSF of RdRp protein with (N) RMSF of ligand O-desmethyl carvedilol. Ligand contacts are colored with green vertical bars indicating protein residues that interact with the ligands.
